# The mitochondrial and endoplasmic reticulum pathways involved in the apoptosis of bursa of Fabricius cells in broilers exposed to dietary aflatoxin B_1_

**DOI:** 10.18632/oncotarget.11321

**Published:** 2016-08-17

**Authors:** Shibin Yuan, Bangyuan Wu, Zhengqiang Yu, Jing Fang, Na Liang, Mingqiang Zhou, Cheng Huang, Xi Peng

**Affiliations:** ^1^ Department of Wild Animal disease, College of Life Science, China West Normal University, Nanchong 637009, Sichuan, The People's Republic of China; ^2^ Key Laboratory of Southwest China Wildlife Resources Conservation (China West Normal University), Ministry of Education, Nanchong 637009, Sichuan, The People's Republic of China; ^3^ Department of Animal Pathlogy, College of Veterinary Medicine, Sichuan Agricultural University, Ya'an 625014, Sichuan, The People's Republic of China

**Keywords:** aflatoxin B_1_, bursa of Fabricius, apoptosis, oxidative stress, broiler

## Abstract

Aflatoxin B_1_ (AFB_1_), a toxic metabolite produced by some fungi, exerts well-known hepatocarcinogenic and immunosuppressive effects, the latter can increase the apoptotic immune cells *in vitro*. However, it is largely unknown that which signaling pathways contribute to excessive apoptosis of immune cells which induced by AFB_1_. In this study, we investigated the roles of the mitochondria, endoplasmic reticulum (ER) and death receptor activated apoptotic pathways in the bursal of Fabricius (BF) cells in the broilers exposed to AFB_1_ diet. We found that (1) AFB_1_ diet induced morphological changes in the BF. (2) FCM and TUNEL methods showed that excessive apoptosis could be resulted from AFB_1_ intake. (3) AFB_1_-induced apoptosis of bursal cells involved mitochondrial pathway (increase of Bax, Bak, cytC, caspase-9, Apaf-1, caspase-3 and decrease of Bcl-2 and Bcl-xL) and ER pathway (increase of Grp78/Bip, Grp94 and CaM). (4) Oxidative stress was confirmed in the BF of chicken fed on AFB_1_ diet. Overall, this work is the first to demonstrate that the activation of mitochondria and ER apoptosis pathways can lead to excessive apoptosis in BF cells, and oxidative stress is a crucial driver during AFB_1_ exposure.

## INTRODUCTION

Aflatoxins (AFs), kind of widespread contaminants of foods and feeds, are toxic metabolites produced by *Aspergillus flavus* and *A. parasiticus*. Among the four major types of AFs (Aflatoxin B_1_, B_2_, G_1_, G_2_), Aflatoxin B_1_ (AFB_1_) is the most potent carcinogen, due to its demonstrated toxic and carcinogenic effects in livestock, poultry and its acute toxicological or chronic hepatocarcinogenic effects in humans [1, 2]. To exert these toxic effects, AFB_1_ requires to convert to AFB_1_-8,9-exo-epoxide (AFBO) [3]. In livestock and laboratory animals, negative effects associated with AFB_1_ exposure include growth retardation and poor feed conversion, increased mortality and leg problems [4–6]. In poultry, decreased reproductive ability, liver and kidney injuries, as well as immunosuppression were observed [7–10]. Moreover, AFB_1_ contaminated rations resulted in the presence of AFB_1_ residues in their edible tissues like liver, muscles and eggs [11, 12], which can potentially create some human health issues [13].

Secondary to the effects on liver, the immunosuppressive nature of AFB_1_ is the best documented area of its toxicity [14], which is related to the increase of disease susceptibility and mortality. Chicken is sensitive to AFB_1._ Low dosages of AFB_1_ may induce obvious immunosuppression, including the decrease of relative weight of immune organs, T-cell subsets, cytokines, antibody titers, complement activity, and pathologic injury of lymphoid tissues [15–18]. Several studies showed that AFB_1_ could induce lesions of immune organs in poultry [19, 20], and it was also suggested that AFB_1_ treatment could induce oxidative stress, cell cycle arrest, excessive apoptosis, and mitochondria injury in lymphoid tissues of chickens in our previous researches [21–23]. These findings indicated that the injuries of immune organs might play critical roles in immunosuppression induced by AFB_1_ administration in chickens, but its mechanisms need to be further clarified.

Apoptosis is a process of programmed cell death that serves as a major mechanism for the precise regulation of cell numbers, and as a defense mechanism to remove unwanted and potentially dangerous cells [24]. It has been reported that the oxidative stress and apoptosis might play key roles in AFB_1_ induced immunotoxicity [22]. Our previous study demonstrated that AFB_1_ treatment caused alteration of Bax, Bcl-2, and caspase-3 expressions, which involved in apoptosis via mitochondrial pathway in broiler's thymus and bursa of Fabricius (BF) [25, 26]. As we know that the mitochondria, death receptor, and endoplasmic reticulum (ER) activated apoptotic pathways are the three key pathways in apoptosis [27]. However, the exact mechanism of AFB_1_ induced BF injury has not been elucidated, and the signaling pathways of AFB_1_ induced BF apoptosis have not been investigated. The current study aimed to examine which apoptotic pathways maybe involved in this apoptotic procedure by using a broiler model.

In this study, we demonstrated that mitochondria and ER activated apoptosis pathways could be triggered in the excessive apoptosis of BF cells in the chickens fed with AFB_1_ diet. The increased mRNA expressions of p53, Bax, Bak, cytC, caspase-9, Apaf-1, caspase-3 and decreased expressions of Bcl-2 and Bcl-xL were related to the activation of mitochondria pathway. And the increased expressions of Grp78/Bip, Grp94 and CaM mean that the ER apoptosis pathway was involved. Decreased antioxidant capacity suggested that oxidative stress might be an important mechanism of excessive apoptosis of BF cells in this study. Our research also found that the mitochondria and death receptor pathways were involved in AFB_1_-induced apoptosis of thymocytes in chicken [28], which was different from BF cells. Its possible reasons were analyzed in this paper too.

## RESULTS

### Relative weight of bursa of Fabricius

**Figure 1:**
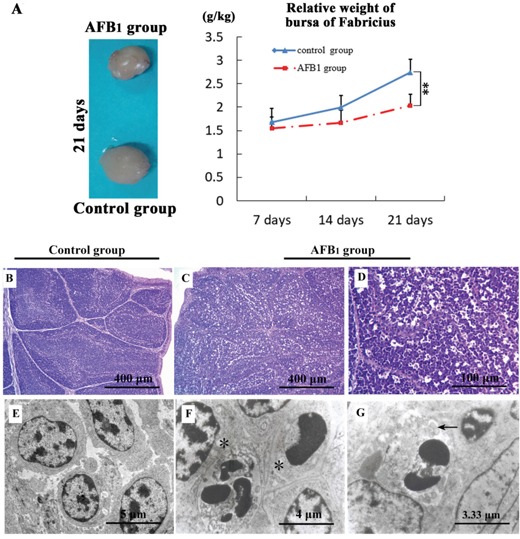
The suppress of aflatoxin B1 exposure on bursa of Fabricius relative weight, histopathology and ultra-structural pathology **A.** The broilers from control and AFB_1_ group, relative weight of BF. Histological assessment of H&E-stained BF tissues from 21-day-old broilers exposed to AFB_1_ diet **C, D.** and control diet **B.** more vacuoles and debris in the lymphoid follicle. Ultrastructural assessment of uranyl acetate and lead citrate stained bursa of Fabricius sections from the control **E.** and AFB_1_ group **F, G.** More apoptotic cells, vacuolated mitochondria with degenerated cristae (*), and expanded ER with high electron density (←) in lymphocytes of BF.

Compared with those of the control group, the relative weight of BF was significantly decreased (p<0.01) at 21 days of age. The results were shown in Figure 1A.

### Histopathological and ultrastructural analysis

Compared with the control group (Figure 1B), there were more vacuoles and debris in the lymphoid follicle of BF in the AFB_1_ group at 7 and 14 days of age, and increased debris were almost in the vacuoles. More vacuoles, more debris and decreased lymphocytes were observed in the lymphoid follicle of BF at 21 days of age (Figure 1C, 1D). Incidence of major histological lesions of BF is shown in Table 1.

**Table 1:** Incidence of major histological lesions of bursa of Fabricius

Pathological Lesions	Time	Control group	AFB_1_ group
Vacuoles increased	7 days	0/6	3/6
	14 days	0/6	4/6
	21 days	0/6	6/6
Nuclear fragmentation increased	7 days	0/6	1/6
	14 days	0/6	4/6
	21 days	0/6	5/6

Incidence of lesions in the BF among animals from different experimental groups (n=6).

In the BF of broilers in the AFB_1_ group, there were some occasional necrotic cells with dilation of perinuclear cisternae, rarefaction of euchromatin and margination of heterochromatin with loss of details. Increased apoptotic cells were characterized as condensed chromatin with petal shape or irregular shape. At the same time, expansive mitochondria with fewer cristae and dilated endoplasmic reticulum are observed in the suspected plasmocytes in the BF. The results are shown in Figure 1F, 1G.

### Apoptotic BF cells analysis by TUNEL assay and flow cytometry method

**Figure 2:**
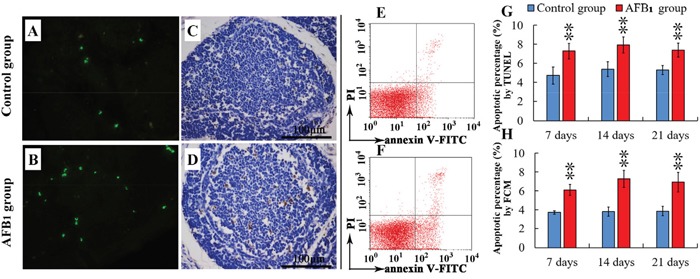
Percentage of apoptotic bursal lymphocytes from the broilers exposed to the control and AFB1 diets TUNEL stained slices of the BF from 21-day-old broilers in the control **A.** and **C.** and AFB_1_ group **B.** and **D.** The nuclei of apoptotic cells were with green fluorescence (stained with FITC fluorescein-dUTP), or brown (stained with diaminobenzidine). Image **E.** and **F.** are representatives of the apoptosis by FCM. Bar graph **G.** and **H.** indicate the mean with standard deviation, and are representatives of apoptosis rate by TUNEL and FCM (*p<0.05, **p<0.01 vs control), six birds per group.

The apoptotic BF cells were stained brown (Figure 2A and 2B) or with green fluorescence (Figure 2C and 2D). The results showed that these apoptotic cells were mostly observed in the medulla of lymphoid follicle. The percentages of apoptotic BF cells in the AFB_1_ group were significantly increased when compared with those of the control group at 7, 14 and 21 days of age (P<0.01). The statistical results are shown in Figure 2G.

Annexin-V-FITC was used to determine the percentage of cells undergoing apoptosis. Apoptotic cells were examined by counting the total percentage of early apoptotic cells (Annexin-V positive and PI negative) and late apoptotic cells (both Annexin-V and PI positive). The results of FCM analysis revealed similar trend as TUNEL. The percentages of apoptotic BF cells in the AFB_1_ group (Figure 2F, 2H) were significantly higher (p<0.01) than those in the control group at 7, 14 and 21 days of age (Figure 2E, 2H), and the statistical results are shown in Figure 2H.

### Changes of Δψm

**Figure 3:**
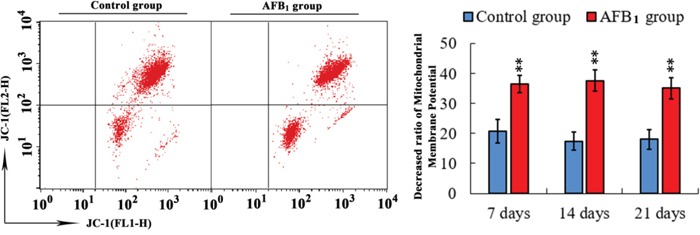
Determining mitochondrial membrane potential in bursa of Fabricius from the broilers exposed to the control and AFB_1_ diets Assessment of mitochondrial membrane potential of BF cells with JC-1 staining by flow cytometry method. Bar graph indicates the mean with standard deviation, and are representatives of the percentage of bursal cells with lowered red fluorescence (*p<0.05, **p<0.01 vs control), six birds per group.

Apoptosis is frequently associated with depolarization of mitochondrial membrane potential (Δψ_m_), which present as reduced JC-1 fluorescence by flow cytometer. That is, the apoptotic population frequently presents lower red fluorescence signal intensity. The results showed that the number of cells with lower red fluorescence in the AFB_1_ group was significantly higher than that in the control group (p<0.01) at 7, 14 and 21 days of age. The results are shown in Figure 3.

### qRT-PCR analysis of relative expressions of apoptosis- and antioxidative-related genes

**Figure 4:**
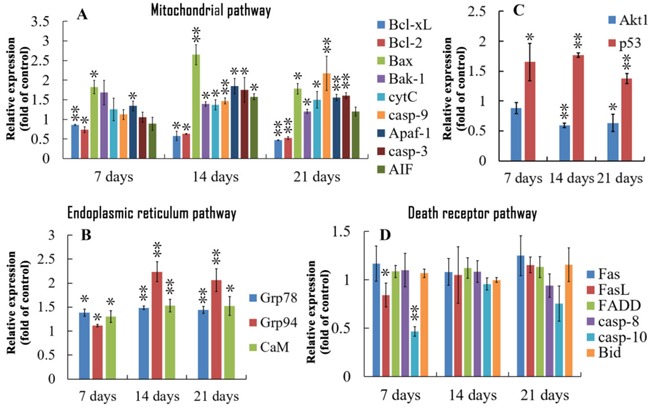
Relative expression of genes regulating apoptosis in bursa of Fabricius from the broilers exposed to the control and AFB_1_ diets **A.** In the mitochondrial pathway, the mRNA expressions of Bcl-xL, Bcl-2, Bax, Bak-1, cytC, casp-9, Apaf-1 and casp-3 in the bursa cells of the AFB_1_-fed broilers are expressed as fold change relative to the control-fed broilers. **B.** In the endoplasmic reticulum pathway, the mRNA levels of Grp78/Bip, Grp94 and CaM in the bursal cells of the AFB_1_-fed broilers are expressed as fold change relative to the control-fed broilers. **C.** The mRNA expressions of p53 and Akt1 in the bursal cells of the AFB_1_-fed broilers are expressed as fold change relative to the control-fed broilers. **D.** In the death receptor pathway, the mRNA expression of Fas, FasL, FADD, casp-8, casp-10 and Bid have no obvious changes compared to the control-fed broilers. All data are expressed as the mean value with deviation. *p<0.05, *p<0.01 vs control, n=6 for each group.

At 7, 14 and 21 days of age, the increased (p<0.05 or p<0.01) mRNA expressions of Bax, apaf-1, Grp78/Bip, Grp94, calmodulin (CaM) and p53, decreased (p<0.05 or p<0.01) expressions of Bcl-xL and Bcl-2 were observed in the BF of the AFB_1_-fed broilers. At 14 and 21 days of age, the mRNA expressions of Bak-1, cytC, casp-9 and casp-3 in the BF of the AFB_1_ group were increased (p<0.05 or p<0.01) and akt1 was decreased significantly (p<0.05 or p<0.01) when compared with those of the control group. What's more, the mRNA content of FasL, casp-10 was decreased at 7 days, and the AIF mRNA level was increased at 14 days of age in the AFB_1_ group (Figure 4).

**Figure 5:**
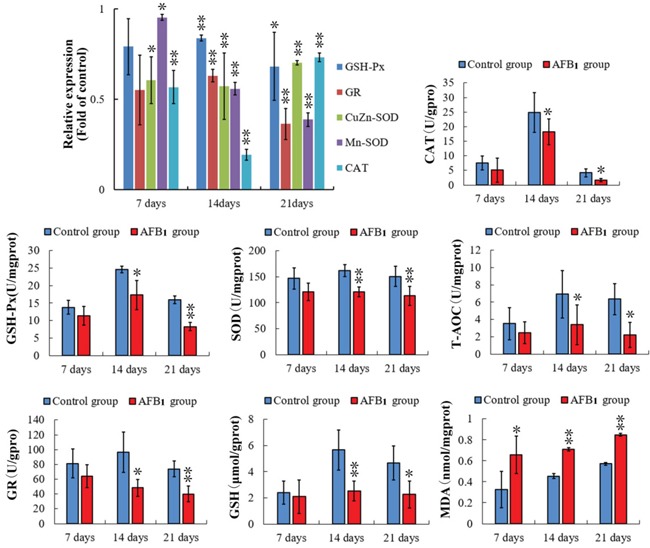
Assessment of the antioxidant function in bursa of Fabricius from the broilers exposed to the control and AFB_1_ diets The mRNA expressions of GSH-Px, GR, CuZn-SOD, Mn-SOD, and CAT in the BF cells of the AFB_1_-fed broilers are expressed as fold change relative to the control-fed broilers. Others bar graph indicate the mean with standard deviation, and are representatives of the enzymatic activities of GSH-Px, GR, CuZn-SOD, Mn-SOD, CAT and T-AOC, and the contents of GSH and MDA (*p<0.05, **p<0.01 vs control), six birds per group.

Comparing with those of the control group, the mRNA expressions of CuZn-SOD, Mn-SOD and CAT were significantly decreased (p<0.05 or p<0.01) in the AFB_1_ group at 7, 14 and 21 days of age. What's more, the mRNA contents of GSH-Px and GR were obviously lower (p<0.05 or p<0.01) at 7 and 21 days of age than those in the control group (Figure 5).

### Antioxidation biochemistry analysis parameters

At 14 and 21 days of age, the activities of CAT, GSH-Px, SOD, GR and T-AOC were significantly lower (p<0.01 or p<0.05) than those in the control group. Comparing to the control group, the decreased (p<0.05 or p<001) contents of GSH and increased (p<0.05 or p<0.01) contents of MDA were observed in the AFB_1_ group at 7, 14 and 21 days of age. The results are shown in Figure 5.

## DISCUSSION

The BF of birds is a specific central immune organ for the proliferation and diversification of B cells, so it is correlated to the humoral immune function. In the present study, dietary AFB_1_ was found to induce the decrease of relative weight, obvious histopathological injury and ultrastructural impairment in the BF of chicken. Previous studies showed that increased vacuoles, nuclear debris and decreased lymphocytes could be a stable histological phenotype of lymphoid tissues [21, 29–31], and increased apoptotic cells and impaired membrane system were observed by ultrastructural examination in AFB_1_-treated broilers [20]. The histopathological and ultrastructural lesions of BF may be one of the main factors of humoral immunosuppression induced by AFB_1_. In this study, dietary AFB_1_ could cause excessive apoptosis of BF cells at early-stage by FCM method and at late-stage by TUNEL assay. Our results were agreed with previous viewpoint [32–34] that the reduced immune function could result from excessive apoptosis of immune cells.

As we know, the mitochondria, ER and death receptor activated apoptotic pathways are the three key pathways in apoptosis [27]. We were therefore interested in discovering which apoptotic pathways play major roles in excessive apoptosis of BF in this study.

The depolarization of Δ***ψ_m_*** was considered to be an early case in mitochondria activated apoptotic pathway [35]. The percentage of BF cells with depolarized Δ*ψ_m_* was increased, and the swelling of mitochondria was induced by AFB_1_ in our study. The mRNA expressions of Bax, Bak, cytC, caspase-9, Apaf-1, caspase-3 were increased and the mRNA expressions of Bcl-2 and Bcl-xL were decreased in the BF, which were correlated to activating of the mitochondrial pathway. The results were in accordance with the changes of corresponding proteins (Bax, Bcl-2 and caspase-3) [26]. Our data suggested that the excessive apoptosis of BF cells may result from the activation of mitochondria activated caspase-bind signaling pathway. Based on the changes of these genes and previous opinions [36, 37], a series of events might occur in the procedure of mitochondria mediated apoptotic pathway: homodimerize of overexpressed Bax, increased permeabilization of mitochondrial outer membrane, release of cytC and ATP from mitochondria, formation of tetramer (composed of caspase-9, Apaf-1, cytC and dATP), autocatalytic activation of caspase-9 and activation of effector caspases including caspase-3. However, in our present study, the relative expression of apoptosis-inducing factor (AIF) had no obvious change when compared with that of the control, suggesting that non-caspase-bind apoptotic pathway was not active in this experiment.

The ER apoptosis pathway is initiated by ER stress, which is due to a number of factors, including cytotoxicity and nutrient limitation, and often characterize as the accumulation of unfolded or misfolded proteins [38, 39]. In the current study, the ER was enlarged with high electron density materials in the suspected plasmocytes, suggesting that unfold protein could be accumulated in ER. The optimum protein folding is decided by several factors including Ca^2+^, ATP and an oxidizing environment [40]. In the present study, the increased mRNA expression of CaM which is important in regulating the concentration of Ca^2+^ [41], ATP deficiency induced by mitochondrial injury and oxidative stress might simultaneously result in unfolded protein response (UPR). The UPR could cause an imbalance of unfolded proteins and chaperones, lastly result in ER stress [42]. During ER stress, proapoptotic members of the Bcl-2 family are recruited to the ER surface, and activate caspase-12, which finally activates caspase-3 [43]. In this study, the increased expression of Grp78/Bip, Grp94 and calmodulin (CaM) showed that ER apoptosis pathway could be involved in the excessive apoptotic of bursal cells.

Furthermore, we investigated the relative mRNA expressions of genes correlated with death receptor apoptotic pathway. The results showed that the expressions of Fas, FasL, FADD, caspase-8, caspase-10 were no difference between the AFB_1_ group and the control group. The results evidenced that death receptor apoptotic pathway may not contribute to the excessive cell death of BF cells.

It is well accepted that oxidative stress is an apoptosis inducer [44, 45]. An imbalance between ROS and the antioxidant may lead to the alteration of structure and function of proteins, lipids, and DNA, and then induce the damage of lipid membranes, cellular catalytic reactions, and finally cell apoptosis [46]. In the current study, the decreased activities of GSH-Px, SOD, CAT, GR, T-AOC, increased concentration of MDA, decreased content of GSH and decreased mRNA levels of antioxidant enzymes (GSH-Px, SOD, CAT and GR) were found in the BF from broilers fed with AFB_1_ diet. Our results suggested that oxidative stress may be a main mediator of excessive apoptosis of BF cells in this study.

**Figure 6:**
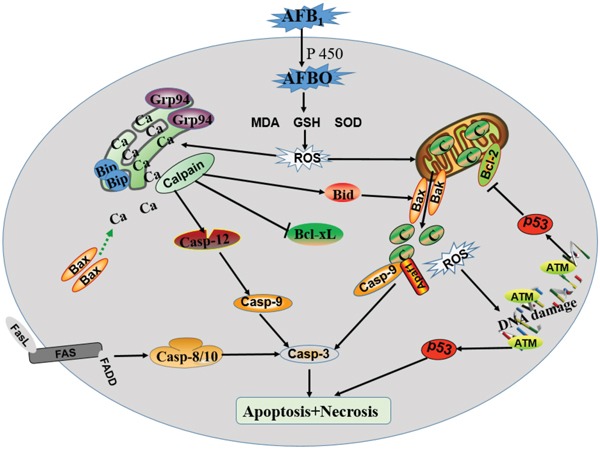
Schematic diagram of the proposed mechanisms of AFB1 induced apoptosis of BF cell A proposed model of BF cells apoptosis by dietary AFB_1_ among mitochondria, endoplasmic reticulum and cell death receptor signaling pathways. ROS can induce mitochondria, endoplasmic reticulum stress, and DNA damage, and subsequently active the apoptosis signaling pathways.

Taken together, our results showed that dietary AFB_1_ exposure are able to induce excessive apoptosis of BF cells in chickens by triggering mitochondria and ER mediated apoptotic pathways (Figure 6), and oxidative stress may be the main factor being responsible for the activation of the two apoptosis pathways. Future studies will focus on a deeper understanding of the mechanisms of AFB_1_-induced immunosuppression. In the same research, we found an interesting phenomenon that different apoptosis-related signaling pathways were involved in the thymocytes and BF cell in chicken. Mitochondria and death receptor pathways were involved in thymocytes in chicken exposed to AFB_1_ [28], but Mitochondria and ER pathways in BF cells, suggesting that toxicological mechanisms were different in thymus and BF. So we can do further contrastive research to clarify this difference.

## MATERIALS AND METHODS

### Chickens and diets

One hundred and fifty-six one-day-old healthy Cobb-500 broilers were purchased from Chia Tai Group (Wenjiang, Sichuan, China), and were randomly divided into two equal groups of three replicates and 26 birds per replicate, namely control group (0 mg/kg AFB_1_) and AFB_1_ group (0.6 mg/kg AFB_1_). The basal diet, namely the control diet, was formulated according to National Research Council (NRC, 1994) [47] and Chinese Feeding Standard of Chicken (NY/T33-2004) recommendations. The AFB_1_-contaminated diet was made, referring to the method described by Kaoud [48]. Briefly, 27 mg AFB_1_ (A6636, Sigma-Aldrich, USA) was dissolved into 30 ml methanol, and then the 30 ml mixture was mixed into 45 kg corn-soybean basal diet to formulate the AFB_1_-contaminated diet. The equivalent methanol was mixed into corn-soybean basal diet to produce the control diet. Then the methanol of diets was evaporated at 98 °F (37 °C). The AFB_1_ concentrations were analyzed by HPLC with fluorescence detection (Waters Model 2475), and the AFB_1_ concentration were determined as <0.001 mg/kg and 0.601 mg/kg respectively in the control diet and AFB_1_ diet. Broilers were housed in cages with electrically heated units and provided with water as well as aforementioned diet ad libitumfor 21 days. The animal protocols and all procedures of the experiment were performed in compliance with the laws and guidelines of Sichuan Agricultural University Animal Care and Use Committee (Approval No: 2012-024). The Ethics Committee for Animal Experiments (Institute of Animal Diseases & Environmental Hazards of Sichuan Province, Chengdu, China) approved our experimental protocol.

### Relative weights of thymus and bursa of Fabricius

At 7, 14, and 21 days of age, after the body weight was recorded, six birds in each group were euthanized and necropsied. Then the BF dissected from each bird and weighed after dissecting connective tissue around them. Relative weight of BF was calculated by the following formula:

Relative weight=organ weight (g)/body weight (kg)

### Histopathological and ultrastructural examination

Six broilers in each group were euthanized at 7, 14 and 21 days of age. The BF were fixed in 4% paraformaldehyde (PFA) and routinely processed in paraffin. Thin sections (5 μm) of each tissue were sliced and mounted on glass. Slides were stained with hematoxylin and eosin Y. The histological structures of the tissues were observed and photographed with a digital camera (Nikon, eclipse 50i, Japan).

At the end of the trial, one chick per replicate in each group was euthanized and then immediately necropsied. Small pieces of BF tissues were rapidly fixed with 2.5 % glutaraldehyde and post-fixed in 2% Veronal acetate-buffered OsO_4_. After dehydration in graded alcohol, the tissues were embedded in Araldite. The blocks were sectioned in a microtome with a glass knife. Sections, 65-75 nm thick, were placed in uncoated copper grids. The sections were stained with uranyl acetate, and post-stained with 0.2% lead citrate. The subcellular structure of BF was examined with a Hitachi H-600 electron microscope (Japan).

### Annexin V-FITC/PI double staining assay

The BF cells of six birds in each group were sampled to determine the apoptosis rate by flow cytometry method at 7, 14 and 21 days of age. Bursal cell suspension was prepared by gently cutting with scissors, and filtered through a 300-mesh nylon screen. Then the cells were washed and suspended in phosphate buffer (PBS, PH: 7.2) at a concentration of 1×10^6^ cells/mL. 100 μL cell suspension was transferred to another centrifuge tube, and stained with 5 μL of Annexin V-Fluorescein isothiocyanate (V-FITC) (BD Pharmingen, USA, 51-65874X) and 5 μL of Propidium iodide (PI) (BD Pharmingen, USA, 51-66211E) for 15 min at 25 °C in the dark. Afterwards, 450 μL of 1×Annexin binding buffer (BD Pharmingen, USA, 51-66121E) was added to the mixture, and the percentages of apoptotic cells were assayed by FCM within 1 hour.

### TUNEL assay

The DNA fragmentation indicative of apoptosis was examined using terminal deoxynucleotidyl transferase-mediated dUTP nick end labeling method (TUNEL). TUNEL assay was performed using Insitu Cell Death Detection Kit (Cat. NO. 11684817910, Roche Molecular Biochemicals, Germany) according to the instructions of the manufacturer, as described by Tayman [49]. Briefly, slices were rehydrated in a series of xylene and ethanol solutions and then incubated in a humidified chamber at room temperature for 20min with proteinase K. Slices were then rinsed with tris-buffered saline (TBS). The entire specimens were covered with 3% H_2_O_2_ and then incubated at room temperature for 5min. Slices were rinsed with TBS. TUNEL enzyme and label solution were mixed and applied to slices, which were incubated again in the humidified chamber for 1h at 37 °C. Slices were thoroughly rinsed with TBS. Stop buffer, block buffer, and conjugate were applied in turn. Diaminobenzidine solution was applied for 10-15min to stain the nuclei of apoptotic cells. The hematoxylin was used to counter-stain the nuclei of normal cells. Slices were dehydrated in a series of three ethanol baths and twice xylene baths, 5min for each. The nuclei of apoptotic cells were with green fluorescence (stained with FITC fluorescein-dUTP), or brown (stained with diaminobenzidine). The TUNEL positive cells (apoptotic cells) were counted using a computer-supported imaging system connected to a light microscope (OlympusAX70) with an objective magnification of ×400. Then apoptotic cells were quantified by Image-Pro Plus 5.1 (USA) image analysis software. Five sections in each group and five fields in each section were measured and averaged.

### Detection of mitochondrial membrane potential (Δψm)

JC-1 (Cat.No.551302, BD, USA) was used to determine mitochondrial membrane potential (Δ*ψ_m_*). A total of 1 mL cell suspension (made in procedure Annexin V-FITC/PI double staining assay) was transferred into 5 mL culture tube and centrifuged. Afterwards, 0.5 ml of JC-1 working solution was immediately added and gently mixed. And then the mixture was incubated for 15 min at 37 °C under 5% CO_2_ incubator. At the end of the incubation, cells were washed twice with 1× Assay Buffer cells, and then re-suspended in 450 μL 1× Assay Buffer. And then Δψm was assayed by FCM within 30 minutes.

### Quantitative real-time PCR (qRT-PCR)

At 7, 14, and 21 days of age, bursas of Fabricius from six birds in each group were removed and immediately stored in liquid nitrogen. Then, bursas of Fabricius were homogenized by crushing with a mortar and pestle. The powdered tissues were collected into eppendorf tubes and stored at −80°C. Total RNA was extracted from BF using TriPure Isolation Reagent (Cat No. 11667165001, Roche Applied Science, Germany) following the procedure provided by manufacturer. The yield of extraction was assessed by measuring light absorbency at 260 nm, and the quality of RNA was detected by calculating the ratio of the absorbency at 260 and 280 nm. Extracted RNA immediately reverse-transcribed into cDNA by using Transcriptor First Strand cDNA Synthesis Kit (Cat No: 04897030001, Roche Applied Science, Germany), according to the manufacturer's instructions. And then the cDNA was used as a template for quantitative real-time PCR analysis.

For qRT-PCR reactions, 20 μL mixtures were made by using FastStart Universal SYBR Green Master mix (Cat No: 04913914001, Roche Applied Science, Germany) containing 10 μL faststart universal SYBR green master (ROX), 0.6 μL forward primer, 0.6 μL reverse primer, 6.8 μL RNAase-free water and 2 μL cDNA. Reaction conditions were set to 10 min at 95 °C (first segment, one cycle), 10 s at 95 °C and 30 s at melting temperature (T_m_) of a specific primer pair (second segment, 44 cycles) followed by 10 s at 95 °C, and 72 °C for 10 s (dissociation curve segment) using Thermal Cycler (Step One Plus, Applied BioSystems, USA). Gene expression was analyzed, and β-actin was used as an internal control [50, 51]. Sequence of primers was obtained from GenBank of NCBI. Primers were designed with Primer 5 and synthesized by Sangon Biotech (Shanghai, China) (Table 1). The qRT-PCR data were analyzed with 2^−ΔΔCt^ calculation method described by Livak and Schmittgen [52].

### Biochemical analysis

At 7, 14 and 21 days of age, six broilers in each group were euthanized and immediately necropsied. Then bursas of Fabricius were immediately removed and put into 0 °C 0.85% NaCl solution. One gram bursa of Fabricius was homogenized with 9 mL 0.85% NaCl solution. After the homogenates were centrifuged at 3500×g at 4°C, the total protein in the supernatant was determined by total protein quantification kit (A045-2). The activities of SOD (A001-1), CAT (A007), GSH-Px (A005) and GR (A062), contents of GSH (A006) and MDA (A003-2), and total ant-oxidative capacity (T-AOC) (A015) in the supernatant were detected using commercial kits (NJJCBIO, Nanjing, China), according to the manufacturer's instructions.

### Statistical analysis

The significance of difference between two groups was analyzed by variance analysis, and results are expressed as the mean value with deviation. The analysis was performed using the independent sample *t* test of SPSS software for Mac v.20.0 (IBM Corp, Armonk, NY, USA) and a value of *p* < 0.05 was considered significant, while *p* value < 0.01 was considered markedly significant.

**Table 2:** A list of oligonucleotides used as primers in qRT-PCR analysis of gene expression in chicken bursa of Fabricius cells

Gene symbol	RefSeq mRNAnumber	Forward primers	Reverse primers	Amplicon length (bp)
GSH-Px	NM001277853	TTGTAAACATCAGGGGCAAA	TGGGCCAAGATCTTTCTGTAA	140
CuZn-SOD	NM205064	CGCAGGTGCTCACTTTAATCC	CTATTTCTACTTCTGCCACTCCTCC	119
Mn-SOD	NM204211	CACTCTTCCTGACCTGCCTTACG	TTGCCAGCGCCTCTTTGTATT	146
GR	GQ853055	CTGTGGCAAAGCCCTCCTGA	ATGGGTGGGTGGCTGAAGAC	135
CAT	NM001031215	CTGTTGCTGGAGAATCTGGGTC	TGGCTATGGATGAAGGATGGAA	160
Bcl-2	NM_205339	TGTTTCTCAAACCAGACACCAA	CAGTAGGCACCTGTGAGATCG	205
Bcl-xl	NM_001025304	GAGGTACCGGAGGGCTTTCA	CAAAGCTCTGGTACGCCGTG	74
Bak-1	NM_001030920	CTGTTCGCTTCCTTCCCCTG	TTGCAGAGATGCTGTGGGAC	167
Bax	XM_422067	GGTGACAGGGATCGTCACAG	TAGGCCAGGAACAGGGTGAA	108
Cyt c	K02303.1	AGGCAAGCACAAGACTGGA	CTGACTATCACCAAGAACCACC	150
Apaf-1	XM_416167	ACCTTTCCCGTCTGGTTGTTC	AGCAATCTCTCTCCGCTTTCT	139
Casp-9	AY057940	CCAACCTGAGAGTGAGCGATT	GTACACCAGTCTGTGGGTCGG	87
AIF	NM_001007490	CTGGGTCCTGATGTGGGCTAT	TGTCCCTGACTGCTCTGTTGC	123
Casp-3	NM_204725	TGGCCCTCTTGAACTGAAAG	TCCACTGTCTGCTTCAATACC	139
Fas	NM_001199487	TCCACCTGCTCCTCGTCATT	GTGCAGTGTGTGTGGGAACT	78
FasL	NM_001031559	GGCATTCAGTACCGTGACCA	CCGGAAGAGCACATTGGAGT	78
FADD	XM_421073	GGGGTAAAGAGGCTGAACTCTTA	TGAGTCCTATTGCACTGCTGTC	163
Casp-8	NM_204592	GTCTCCGTTCAGGTATCTGCT	TCTCAATGAAAACGTCCGGC	143
Casp-10	XM_421936	CTGGGGGCTCCAAAAGTCC	AAAGGGGGACAAAGCCAACA	204
Bid	NM_204552	GAGCAGCTTGCTGGAGAGAA	GAGGCAGCTGGATCACAAGT	187
Grp78	NM_205491	GGTGTTGCTTGATGTGTGTCC	GCTGATTGTCAGAAGCTGTGG	134
Grp94	NM_204289	TGACCTGGATGCAAAGGTGGA	TTAAACCCCACACCATCCCTCAAC	250
CaM	NM_205005	GGAGTTGGTAAAATGAGGGAACA	ACATTGTGGACGATTGACAGTCT	233
p53	NM_205264.1	ACCTGCACTTACTCCCCGGT	TCTTATAGACGGCCACGGCG	127
Akt1	NM_205055.1	AGGCAGCCTCCTCCTCTC	GGCTCCTCCTCCCCTTCTC	114
β-actin	L08165	TGCTGTGTTCCCATCTATCG	TTGGTGACAATACCGTGTTCA	178
